# Carbon nanoparticles induce ceramide- and lipid raft-dependent signalling in lung epithelial cells: a target for a preventive strategy against environmentally-induced lung inflammation

**DOI:** 10.1186/1743-8977-9-48

**Published:** 2012-12-10

**Authors:** Henrike Peuschel, Ulrich Sydlik, Susanne Grether-Beck, Ingo Felsner, Daniel Stöckmann, Sascha Jakob, Matthias Kroker, Judith Haendeler, Marijan Gotić, Christiane Bieschke, Jean Krutmann, Klaus Unfried

**Affiliations:** 1 IUF Leibniz-Institut für Umweltmedizinische Forschung, Auf'm Hennekamp 50, 40225, Düsseldorf, Germany; 2 Department of Material Chemistry Institute Ruđer Bosković, Zagreb, Croatia

**Keywords:** Carbon black, Air pollution, Epidermal growth factor receptor, c-src, Alpha-tocopherol, Ectoine, compatible solutes

## Abstract

**Background:**

Particulate air pollution in lung epithelial cells induces pathogenic endpoints like proliferation, apoptosis, and pro-inflammatory reactions. The activation of the epidermal growth factor receptor (EGFR) is a key event responsible for signalling events involving mitogen activated protein kinases specific for these endpoints. The molecular events leading to receptor activation however are not well understood. These events are relevant for the toxicological evaluation of inhalable particles as well as for potential preventive strategies in situations when particulate air pollution cannot be avoided. The current study therefore had the objective to elucidate membrane-coupled events leading to EGFR activation and the subsequent signalling cascade in lung epithelial cells. Furthermore, we aimed to identify the molecular target of ectoine, a biophysical active substance which we described to prevent carbon nanoparticle-induced lung inflammation.

**Methods:**

Membrane signalling events were investigated in isolated lipid rafts from lung epithelial cells with regard to lipid and protein content of the signalling platforms. Using positive and negative intervention approaches, lipid raft changes, subsequent signalling events, and lung inflammation were investigated in vitro in lung epithelial cells (RLE-6TN) and in vivo in exposed animals.

**Results:**

Carbon nanoparticle treatment specifically led to an accumulation of ceramides in lipid rafts. Detailed analyses demonstrated a causal link of ceramides and subsequent EGFR activation coupled with a loss of the receptor in the lipid raft fractions. In vitro and in vivo investigations demonstrate the relevance of these events for carbon nanoparticle-induced lung inflammation. Moreover, the compatible solute ectoine was able to prevent ceramide-mediated EGFR phosphorylation and subsequent signalling as well as lung inflammation in vivo.

**Conclusion:**

The data identify a so far unknown event in pro-inflammatory signalling and contribute to the understanding of particle cell interaction and therefore to risk identification and risk assessment of inhalable xenobiotics. Moreover, as this cellular reaction can be prevented by the well tolerated substance ectoine, a molecular preventive strategy for susceptible persons against airway inflammation is proposed.

## Background

The carbonaceous fraction of environmental pollution has been described to be the main determinant for adverse health effects due to exposure against airborne xenobiotics [1]. In particular, combustion derived nanoparticles are considered as a major component of particulate matter responsible for the induction and aggravation of diseases in humans [2]. Besides environmental exposure routes, humans may also come in contact with this kind of xenobiotics in the form of carbon nanoparticles (CNP) which are produced in combustion processes for technological applications. Humans are therefore exposed to this kind of nanoparticles in environmental as well as in occupational settings, and exposure through inhalation has been associated with severe outcomes like lung cancer, COPD, and cardiovascular diseases [3].

A main mechanism determining the toxicity of inhaled particles and causative for lung diseases but also for systemic health effects is the inflammation of the airways. As direct reaction on inhaled particles, the release of inflammatory factors like interleukin-8 (IL-8) can be triggered by a direct interaction of particles with lung epithelial cells [4]. In this context, the activation of the epidermal growth factor receptor (EGFR) appears to be a key event responsible for the expression and release of this pro-inflammatory chemokine which is responsible for the recruitment of neutrophils in the lung [5]. The activation of EGFR has been reported to be triggered by air pollutants like ozone, diesel emission particles (DEP) and tobacco smoke but also by pure CNP [6-8].

The release of pro-inflammatory IL-8 from human bronchial epithelial cells has also been correlated to oxidative events [9]. Several studies demonstrated that oxidative stress is also involved in phosphorylation of EGFR and the subsequent activation of signalling cascades in tissues exposed to the environment like alveolar or bronchial epithelia [10,11]. The initial link between nanoparticle-derived reactive oxygen species (ROS) and EGFR signalling, however, is so far not understood.

EGFR is known to be located in functional lipid domains defined either by their protein components like caveolin [12] or by their specific lipid composition like enriched gangliosides [13]. Upon activation, either by ligands or by oxidative stress, membrane rearrangement at the level of lipids as well as of proteins occur and the active receptor becomes internalized while the subsequent signalling cascade is initiated [14,15]. Although some influence of nanoparticles on active mechanisms of uptake and internalization as well as effects on the membrane fluidity have been described [16], it is not clear how nanoparticles interfere with membrane-coupled signalling processes. For CNP, the influence of ROS on membrane-coupled signalling has been demonstrated [8]. However, it had to be demonstrated in which way oxidative stress triggers these molecular events.

Inflammatory reactions of the lung triggered by CNP as environmental model particles can be prevented in vivo by compounds of the group of compatible solutes. Our earlier studies with rat lung epithelium demonstrated that membrane-coupled pro-inflammatory signalling resulting in the release of IL-8 is significantly reduced in the presence of the compatible solute ectoine [17]. More recently, we were able to show that similar signalling events elicited by CNP, but also by pro-inflammatory factors, in neutrophilic granulocytes can be prevented by ectoine [18]. As these events in neutrophils result in the reduction of natural apoptosis, the application of ectoine appears suitable for the treatment of chronic neutrophilic inflammation in diseases like COPD or fibrosis. Compatible solutes are low molecular weight natural compounds which are produced by cells in order to survive under stress conditions like high salt, desiccation, or high temperature. Besides their function to act as osmotic counterpart, these substances have been described to stabilize macromolecules and to have an impact on membrane fluidity [19-21]. The compatible solute ectoine so far has been shown to prevent signalling events triggered by UVA irradiation in skin epithelial cells. This kind of environmental stress initiates pro-inflammatory signalling via the induction of ROS in keratinocytes [22]. As a consequence of this oxidative stress, ceramides are generated by non-enzymatic degradation of sphingomyelin as an immediate early reaction which is followed by de novo synthesis of ceramides. In this system, ectoine prevented the increase of ceramides by UVA radiation within lipid rafts [23].

In the current work, we aimed to investigate molecular mechanisms of CNP-induced EGFR activation linking the particle-associated oxidative stress and pro-inflammatory signalling. Using the compatible solute ectoine as a substance which is not considered to act as an anti-oxidant but is known to interfere in a biophysical way with lipid raft signalling [23], we asked whether CNP specifically (compared to non-nano carbon particles, CP) induce changes in membrane composition that result in the activation of EGFR. For this purpose, detergent resistant membranes (DRM) from lung epithelial cells treated with either CNP or CP were isolated and analyzed for their lipid and protein composition. Such changes at the level of lipid rafts were causally linked to signalling pathways responsible for pathogenic endpoints which we identified in vivo in animal experiments. Moreover, the molecular mechanisms for the preventive effect of ectoine on membrane-linked signalling events were investigated in the current study.

## Results

### CNP increase ceramide levels in lipid raft membrane fractions

**Figure 1:**
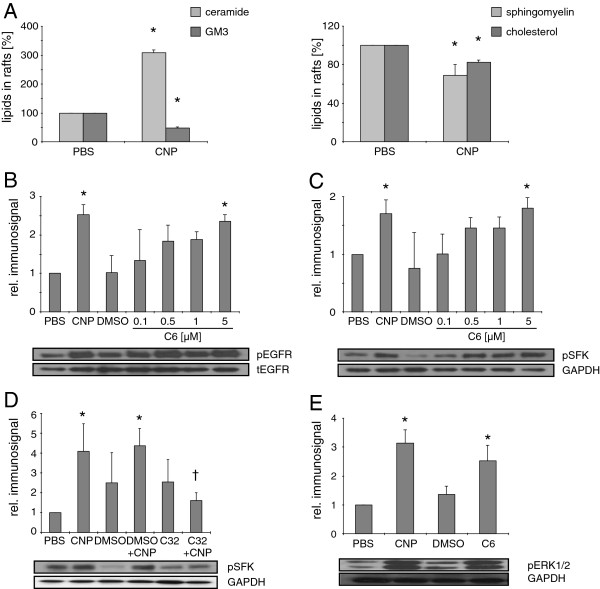
**CNP trigger lipid changes in rafts and subsequent EGFR and SFK activation.** RLE-6TN cells were incubated with CNP [10 μg/cm^2^, 5 min] or the indicated doses of C6 ceramide [μM, 15 min] respectively. (**A**) Amount of ceramide, GM3, sphingomyelin, and cholesterol was detected after HPTLC. (**B**) Phosphorylation of EGFR at Tyr^1173^, (**C** + **D**) SFK at Tyr^416^, and (**E**) ERK1/2 determined by Western Blot using phospho-specific antibodies. Equal loading was confirmed with GAPDH or total EGFR. C32, compound 32, * significantly different from respective control, † significantly different from CNP-treatments without C32 pre-treatment [10 μM, 1 h].

In a first approach, possible changes in the lipid composition of detergent resistant membrane (DRM) fractions, as equivalents of lipid rafts, triggered by CNP in epithelial cells (RLE-6TN) were studied. Treatment of lung epithelial cells within minutes leads to an activation of membrane-coupled signalling cascades involving src family kinases (SFK) and mitogen activated protein kinases (MAPK; 25). Therefore, initial events at the level of the membrane were investigated in DRM isolated by density centrifugation from cells treated with particles for 5 minutes. The particle dose of 10 μg/cm^2^ has been chosen as an equivalent of a cumulative environmental exposure dose which has been shown earlier to induce MAPK activation in lung epithelia in vitro. Such treatment, however, does not induce cytotoxic effects (Additional file 1: Figure S1). As an early response on the particle treatment, in high performance thin layer chromatography (HPTLC) analyses (Figure 1A) the ceramide content was increased while the amounts of ganglioside GM3 and sphingomyelin were significantly decreased. This decrease was accompanied by a loss of cholesterol in lipid rafts.

### Phosphorylation of EGFR and SFK is specifically triggered by CNP and ceramide

In a next set of experiments the possible key function of ceramides for CNP-triggered signalling was tested in lung epithelial cells treated with CNP [10 μg/cm^2^ or increasing doses of externally added C6 ceramide. Both treatments led to the activating phosphorylation of EGFR at position Tyr^1173^ (Figure 1B). The dose dependent increase in Tyr^1173^ phosphorylation proved to be significant in a correlation analysis (Pearson, r=0.58, p=0.004). In order to link this reaction with membrane coupled signalling events which are supposed to be involved in pathogenic EGFR signalling, we further on investigated the effects of ceramide on the activation of SFK and the MAPK Erk1/2. The described cell treatments resulted in significant increases of the activating phosphorylation of SFK at tyrosine residue 416 (Tyr^416^), both, by ceramide (Pearson correlation, r=0.65, p=0.002) and CNP (Figure 1C). In an additional experiment using the pharmacological EGFR inhibitor *compound 32*, the causal connection of EGFR activity and SFK phosphorylation was demonstrated by a significant reduction of SFK phosphorylation in cells treated with CNP in the presence of the inhibitor (Figure 1D). As the central signalling step causally linked to EGFR and SFK activation [24,25] Erk1/2 phosphorylation was investigated (Figure 1E). Again the application of C6 ceramide [5 μM] led to an increase in Erk1/2 phosphorylation which was comparable to the reaction in response to CNP, demonstrating the ability of nanoparticle-triggered ceramide to induce this signalling step. Together with earlier results, the data identify EGFR as the most upstream protein component of the described nanoparticle specific signalling pathway activated by ceramides.

### EGFR and SFK disappear from lipid rafts after activation by CNP and ceramide

**Figure 2:**
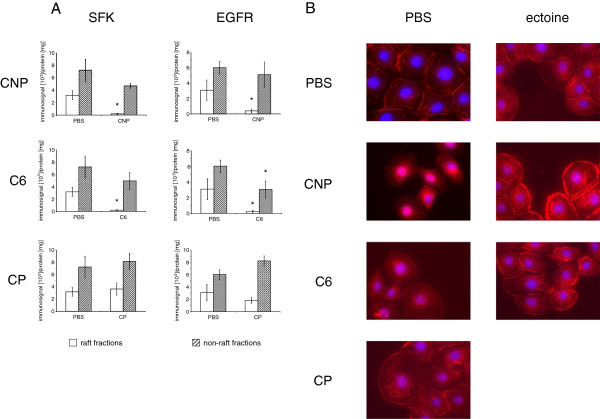
**CNP induce loss of SFK and EGFR from lipid rafts.** (**A**) RLE-6TN cells were treated with CNP [10 μg/cm^2^, 5 min], C6 [5 μM, 15 min], or CP [10 μg/cm^2^, 5 min] and raft and non-raft fractions were isolated and pooled according to the scheme depicted in the supplementary material. Western Blot analyses using total SFK or EGFR antibodies were performed. *, Significantly different to PBS control (p < 0.05). (**B**) RLE-6TN cells were treated with CNP [10 μg/cm^2^, 5 min], CP [10 μg/cm^2^, 5 min], ectoine [1 mM, 4 h pre-treatment] + CNP, or C6 [5 μM, 15 min]. EGFR (red) localization was analyzed by immunostaining and fluorescence microscopy. Nuclei were stained with DAPI (blue).

In order to investigate the influence of CNP on signalling events arising in lipid rafts, DRM from differently treated cells were analyzed for the specific occurrence of signalling proteins by Western blotting of the respective raft and non-raft fractions (Figure 2A). The treatment of cells with particles or control substances had no effect on the integrity of lipid rafts (Additional file 1: Figure S2). Despite the lack of difference in the total protein amount, the signalling proteins SFK and EGFR were significantly reduced in raft fractions from cells treated with CNP, indicating the activation and translocation of the EGFR signalling complex. Importantly, such an event was not observed when the cells were treated with similar mass doses of CP, indicating the specificity of the observed reactions for nanoparticles or for materials with a very high surface reactivity. As a proof of principle for the described signalling events, the translocation of SFK and EGFR in cells treated with C6 ceramide [5 μM] was also observed. The cellular fate of EGFR after activation was investigated microscopically after EGFR immunostaining (Figure 2B). While in the control cells a clear membrane localization of EGFR was observed, the treatment with CNP or with C6 ceramide resulted in an internalization of the receptor. The treatment with the non-nano CP had no effect on the EGFR translocation.

In this set of experiments (Figure 2B), the preventive effect of the compatible solute ectoine on the activation of EGFR was also tested. Earlier studies have shown that ectoine effects are very unlikely to be mediated by changes in particle properties [17]. Pre-treatment of the cells with 1 mM ectoine clearly reduced the translocation of the receptor either induced by CNP or by ceramide. Therefore, the current data indicate that very early reactions are prevented by this compatible solute at the level of membrane-coupled signalling.

### EGFR and SFK signalling in vivo

**Figure 3:**
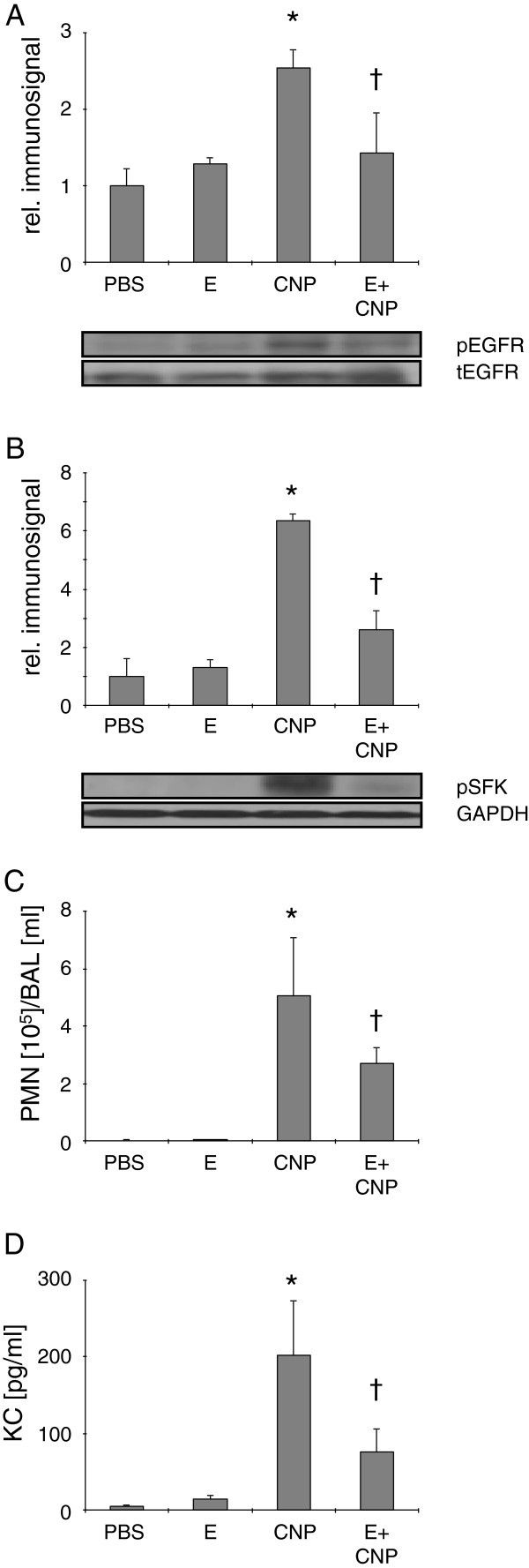
**Ceramide-dependent signalling steps are associated with lung inflammation in mice.** Activation of signalling steps in lung homogenate and induction of inflammation determined in BAL in mice (n = 5) treated with PBS, CNP [5 mg/kg bodyweight], or CNP together with ectoine [E, 1 mM]. (**A**) Phospho-EGFR at Tyr^1173^ signals relative to total EGFR signals. (**B**) Phospho-SFK at Tyr^416^ signals relative to GAPDH signals. (**C**) Neutrophilic granulocytes in BAL (**D**) KC in BAL. *, Significantly different from control; †, significantly different from CNP alone (p < 0.05).

The physiological relevance of the so far identified membrane-dependent signalling was next investigated in vivo in animal experiments in which C57BL/6 mice were exposed to CNP suspensions via pharyngeal aspiration (Figure 3). After 24 h a significant increase of EGFR at position Tyr^1173^ was observed in the lung tissue (Figure 3A). Accordingly, SFK phosphorylation (Tyr^416^) occurred in the lungs of the same animals (Figure 3B). These signalling events were reflected by the induction of neutrophilic inflammation measured by the percentage of neutrophils per ml lavage (Figure 3C) but also by the increase of neutrophil recruiting chemokine KC (IL-8 equivalent in mice; Figure 3D). As an intervention strategy for the identified signalling triggered by nanoparticle derived ceramide, 1 mM ectoine was applied together with CNP. This treatment was able to significantly reduce pathogenic cell stress events at the level of phosphorylation of signalling proteins and consequently reduced lung inflammation determined in BAL.

### Ceramide-triggered signalling depends on ROS and can be prevented by ectoine

EGFR activation and subsequent signalling cascades have been correlated with CNP-induced intracellular oxidative stress [8]. The next set of experiments, therefore, aimed to investigate the link of CNP-induced lipid raft signalling and ROS. Moreover, the identification of the molecular targets of ectoine was intended.

**Figure 4:**
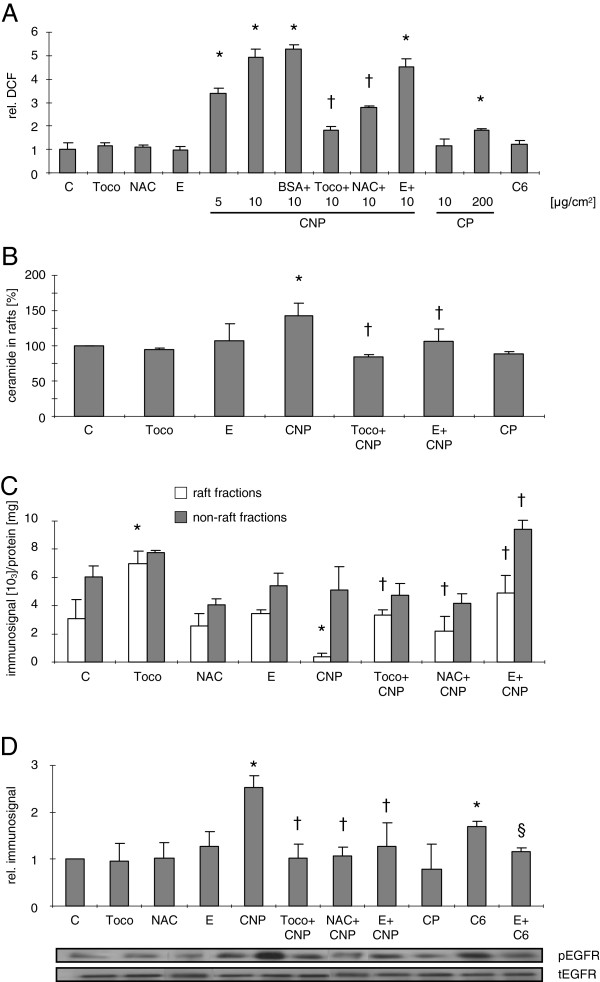
**Ectoine as well as antioxidants prevent ceramide generation and subsequent receptor translocation.** (**A**) Flow cytometric analysis of intracellular ROS generation in RLE-6TN cells measured by relative intracellular DCF fluorescence after treatment with the indicated doses of CNP [μg/cm^2^, 1 h], CP [μg/cm^2^, 1 h], BSA-coated CNP, or C6 ceramide [5 μM, 15 min] and pre-treatment with the respective inhibitors tocopherol [Toco; 50 μM] N-acetylcysteine [NAC; 0.1 mM], or ectoine [E; 1 mM]. C indicates respective vehicle controls. (**B**) Amount of ceramide generation was detected by lipid extraction and HPTLC in RLE-6TN cells. (**C**) EGFR translocation as described in Figure 2 after treatment and pre-treatment as described above. (**D**) EGFR activation as described in Figure 1 after treatment and pre-treatment as described above. Means in **A**, **C**, and **D** are given as values relative to the respective controls. *, Significantly different from control; †, significantly different from 10 μg/cm^2^ CNP alone; §, significantly different from C6 alone.

As a first step, intracellular oxidative stress was measured flow cytometrically using dichlorofluoresceine (DCF, Figure 4A). Cell treatment with 5 μg/cm^2^ and 10 μg/cm^2^ CNP resulted in a significant increase of intracellular fluorescence. Exposing cells to an equal mass dose [10 μg/cm^2^] of CP did not lead to elevated ROS levels. An exposure scenario based on equal surface area with CP (200 μg/cm^2^ CP versus 5 μg/cm^2^ CNP) led to a significant ROS induction by CP which, however, was markedly lower than with CNP. In order to control for possible changes in surface properties by particle protein interaction, CNP were pre-incubated with [1 μg/ml] bovine serum albumine prior to cell exposure. This kind of treatment led to a change in the zeta potential of the particles but did not significantly change the particle aggregate size (Additional file 1: Table S1). Protein coated particles also increased DCF fluorescence significantly. Scavenging ROS at the level of the membrane by adding tocopherol [50 μM] or increasing the antioxidant capacity of the cells by pre-incubation with N-acetylcysteine (NAC) [0.1 mM] both significantly reduced CNP-specific DCF fluorescence. Ectoine is not considered as a ROS-scavenging substance and therefore has no influence on CNP-specific intracellular oxidative stress. As ceramides are hypothesized to be generated as a consequence of ROS, the lack of DCF fluorescence in C6-treated cells was expected.

The specificity of the ceramide generation by CNP was investigated in additional HPTLC assays monitoring ceramide contents in lipid rafts (Figure 4B). CP which did also not trigger oxidative stress, had no effect on the ceramide levels of lipid rafts. Accordingly, in cells pre-treated with tocopherol, CNP did not induce increased ceramide levels. Both results corroborate earlier findings that show a ROS-dependent and probably non-enzymatic generation of ceramides [22,23]. Ectoine, however, which has no ROS scavenging properties (Figure 4A), also slightly reduced ceramide levels in lipid rafts. This finding may indicate an unexpected mechanism counteracting ceramide generation which is different from antioxidants.

Further on, the influence of both antioxidant strategies as well as of ectoine on the cellular translocation of EGFR was investigated in raft and non-raft membrane fractions of exposed cells (Figure 4C). The loss of EGFR from raft fractions induced by the CNP treatment, which we describe here as a consequence of ceramide generation, is significantly reduced in cells pre-treated with either antioxidants or ectoine. In this experiment however it can be observed that tocopherol alone has also an influence on the localization of EGFR in the raft fractions. Furthermore, ectoine in CNP-treated cells not only increased the amount of EGFR in the non-raft fractions but also appeared to enhance EGFR stability in the non-raft fractions.

Finally, the link between oxidative stress and subsequent ceramide generation and the activation of EGFR was tested by measuring EGFR phosphorylation in the presence of NAC and tocopherol as quenchers of oxidative stress and ectoine as an inhibitor of ceramide accumulation in the lipid raft (Figure 4D). All intervention strategies led to a reduction of EGFR phosphorylation. In addition, the treatment with CP, again, demonstrates the specificity of this endpoint for nanosize carbon particles.

## Discussion

### Ceramides as components of lipid raft signalling by carbon nanoparticles

The generation of ceramide as an intermediate of sphingolipid metabolism has been suggested to cause pulmonary diseases triggered by xenobiotic stress like cigarette smoke [7]. Ceramides acting as second messenger are able to induce apoptotic processes responsible for the induction or the aggravation of acute lung injury, which may result in emphysema and COPD [26]. The current data, however, are indicative for another mechanism of ceramide action in the pathogenesis of lung diseases which is relevant for exposure to the carbonaceous core fraction of combustion derived nanoparticles. Pure CNP trigger the accumulation of ceramides in lipid raft signalling domains. First results indicating the possibility of such a mechanism came from adenocarcinoma cells in which oxidative stress led to a co-localization of ceramides and activated EGFR and SFK [27]. The authors postulate a rearrangement of lipid rafts mediated by ceramides which is responsible for the activation of EGFR. Our data, in fact, demonstrate that an increase of ceramides in these membrane fractions initially causes the activation of signalling cascades via EGFR in lung epithelium and subsequently is responsible for the induction of neutrophilic lung inflammation in vivo. The induction of this signalling cascade by the externally added C6 ceramide gives evidence that ceramides are key molecules in this xenobiotic-induced adverse signalling.

### Oxidative stress specifically triggered by nanoparticles causes lipid raft signalling

The studies presented here identify the generation of intracellular oxidative stress as the initial event in a cascade of membrane signalling events involving the accumulation of ceramides in lipid rafts and the activation of the membrane-coupled receptor EGFR. Particle properties triggering intracellular ROS, thus, may be considered as determining for the subsequent adverse signalling. Pure CNP are known to generate ROS due to their high surface area and the specific surface reactivity [28]. Interestingly, CP which differ in primary particle size and in the specific surface area per mass unit but appear to form aggregates in the same size range as CNP failed to trigger this reactions when applied as equal mass doses. As the exposure experiments based on equal surface area did not reveal convincing correlation of surface area and ROS generation, we cannot exclude an additional particle characteristic determining this reaction. However, it has to be considered that in order to achieve equal surface area, unphysiologically high mass doses of CP have to be applied. Nanoparticle specific induction of ROS has also been described for other poorly soluble nanoparticles with quite different chemical composition [29]. Our own earlier data indicated that the signalling cascade is also induced by silica nanoparticles [24]. The relevance of the described signalling events for other poorly soluble nanoparticles appears likely and has to be demonstrated. Such investigations will be of particular interest with modern colloidal nanoparticles which may have a low potential for ROS generation. The activation of the EGFR as early event of adverse particle effects has also been reported for diesel particles [9]. In these studies, the main effects have been attributed to the organic fraction of DEP. The particles chosen in our experiments, however, are known to contain low amounts of PAH (polycyclic aromatic hydrocarbons) which are not easily mobilised and may not be responsible for the effects which are observed after minutes [30]. We therefore conclude that the signalling events can be assigned to particulate nature of the applied xenobiotic.

Including recent and earlier data, we are now able to postulate an initial signalling cascade mediating adverse effects of CNP via MAPK. These signalling elements have been described to mediate the endpoints apoptosis, proliferation, and expression of pro-inflammatory cytokines, respectively in lung epithelial cells exposed to doses of CNP which in real life may be achieved by cumulative environmental exposure [17,24,28]. Upstream of MAPK activation, events of membrane coupled signalling have been identified to be specifically elicited by CNP. Involving EGFR and integrins, CNP specifically activate SFK and subsequently the signalling cascade via phosphatidylinositol-3-kinase (PI3-K) and protein kinase B (Akt; [25,31].

### Compatible solutes prevent cell stress-triggered ceramide signalling

Compatible solutes have been described to interact with macromolecules via the biophysical mechanism of preferential exclusion [32]. Due to their mostly zwitter ionic nature they interact with water molecules and indirectly with hydration layers of soluble molecules [33]. It is accepted that proteins in the presence of compatible solutes shift into thermodynamically stable conformations. In vitro investigations on the fluidity of artificial membranes indicate that the presence of these substances significantly changes this parameter [21]. The data of the current study now demonstrate that these biophysical molecular events which were mostly identified in cell free systems are relevant in lung epithelial cells and in the organ system of the lung. While ectoine had no influence on the very initial event of intracellular ROS generation, it had effects on the two subsequent molecular events. (i) the accumulation of ceramides in the lipid rafts after particle stressappears to be reduced in the presence of ectoine, and (ii), more strikingly, the ceramide-triggered phosphorylation and dislocation of EGFR from the lipid rafts were reduced by ectoine as shown by the experiments in which ceramide was added externally. Accordingly, all subsequent signalling events are also prevented by ectoine.

### Ceramide mediated signalling as target of a preventive molecular strategy

The signalling events described here which are mediated by the accumulation of ceramides are of particular relevance for individuals who are chronically exposed to traffic related particulate air pollution. This kind of environmental xenobiotic has been associated with several systemic adverse health effects in humans. In particular, the occurrence of mild forms of COPD was observed in a cohort with increased neutrophilic lung inflammation due to long term exposure to this kind of air pollution [34]. Our own studies demonstrate that lung inflammation dominated by neutrophilic granulocytes which is typical for COPD is triggered by the carbonaceous core of these environmental particles via the activation of MAPK pathways in lung epithelial cells [17]. The in vivo investigations of this study demonstrate that the recently identified signalling events are of physiological relevance and that they contribute to pathogenic outcomes like neutrophilic lung inflammation. This fact is mainly demonstrated by the preventive effect of ectoine on lung inflammation in mice (and rats, as described earlier; 17) and appears to be caused by the preventive effect on the activation of EGFR and SFK in vivo. As nanoparticles of other chemical composition are likely to also trigger the signalling events described in this study, ectoine may also prevent adverse effects of these xenobiotics. Together with our more recent findings showing that ectoine is also therapeutically active at the level of neutrophilic granulocytes themselves [18], the data of the actual study indicate that the application of ectoine in human lungs may be beneficial in situations in which air pollution cannot be reduced by technological measures or, more importantly, for persons suffering from respiratory diseases.

## Conclusions

The data presented here demonstrate the central role of the accumulation of ceramides in the lipid raft fraction of lung epithelial cells for the induction of pro-inflammatory reactions and the development of lung inflammation in response to environmental model particles. The activation of EGFR signalling in the lung by xenobiotics so far was mostly associated with organic compounds or transition metals contaminating the particulate fraction of inhaled air pollution like PM or tobacco smoke. Here we show that the pure carbonaceous fraction of inhaled particles is able to trigger pathogenic responses of the epithelium employing a lipid raft-dependent mechanism. Moreover, the compatible solute ectoine is identified as a substance that specifically prevents the described signalling events. The data therefore provide mechanistic insight into the molecular events relevant for the preventive and/or therapeutic use of compatible solutes.

## Methods

### Particle suspensions

Carbon nanoparticles (CNP, Printex 90) were obtained from Degussa (Germany), and carbon particles (CP) were from H. Haeffner (Chepstow, UK; as Huber 990). Stock suspensions [1 mg/ml] of particles were prepared in PBS by sonication for 60 min. Particles and particle suspensions were characterized by (i) scanning electron microscopy (JSM 7000 F, JEOL Ltd., Japan), (ii) BET using FlowSorb II 2300 analyzer (Micromeritics, Norcross, USA), and (iii) light scattering using ZetratracTM NPA152 (Microtrac, Montgomeryville, PA, USA). Particle properties are listed in Table 1S (see Additional file 1).

### Cell culture and exposure

The rat lung epithelial cell line RLE-6TN was purchased from ATCC (Manassas, VA) and grown at 37°C, 5% CO_2_ in supplemented Ham's F-12 medium [24]. For experiments, cells were grown to 80 – 90% confluence, then starved for 20 h in serum reduced medium [0.5% FCS] prior to particle exposure [10 μg/cm^2^. Cells were treated with CNP and CP for up to 1 h in the absence or presence of inhibitors. Inhibitors were added to the cells 18 h (NAC), 4 h (ectoine), or 60 min (compound 32, alpha-tocopherol) prior to treatment with CNP or C6 ceramide (N-hexanoyl-D-erythro-shingosine, Calbiochem, Schwalbach, Germany). Alpha-tocopherol [50 μM] was solubilized in ethanol (final ethanol concentration 0.03%). C6 ceramide was solubilized in DMSO (final DMSO concentration 0.1%). In experiments using these compounds, respective vehicle controls were performed. The effect of DMSO on lipid raft composition was investigated in one control experiment. DMSO treated samples showed no difference to PBS treated samples.

### Cytotoxiciy assay

Cell viability was evaluated by WST-1 (4-[3-(4-iodophenyl)-2-(4-nitrophenyl)-2H-5-tetrazolio]-1,3-benzene disulfonate) assay (Roche diagnostics, Mannheim, Germany) according to the manufacturers’ instructions after 1 h exposure as described above. Eight-fold measurements were performed in three independent (n=3) experiments. Viability was estimated relative to medium (negative) controls and exposure to NaN_3_ [1 M] as positive control.

### Animal experiments

Female C57BL/6JRj mice (8 weeks old, Janvier, France) were treated via pharyngeal aspiration with a volume of 50 μl suspension, under inhalation anaesthesia (isoflurane, 5%, 1–2 min). Animals were sacrificed by exsanguination under anaesthesia 24 h after treatment. After broncho-alveolar lavage (4 x 1 ml PBS), lung tissues were minced, shock frozen and stored at −80°C until further use. Differential cell counts were performed from Giemsa/May-Grünwald stainings of lavage cells. Cell free lavage fluids were subjected to solid-phase ELISA in order to determine KC (R&D systems, Minneapolis, MN). All animal experiments were performed after relevant permission according to German animal protection laws.

### Isolation of detergent resistant membrane raft fractions

Membrane fractions were isolated by density gradient ultracentrifugation as described (22). Exposed cells were harvested in 1 ml TNE buffer [50 mM Tris/HCl pH 7.4, 150 mM NaCl, 2 mM EDTA]. After Dounce homogenization (25 strokes in 1 ml TNE), Triton X-100 was added (1% w/v final concentration). After 30 min on ice the lysate was subjected to density centrifugation (5-30-50% OptiPrep™, 40 000 rpm, rotor 50.2-Ti, 4 h at 4°C). Fractions (7 × 600 μl) were collected starting at the top of each gradient.

### Dot blot

Identification of raft fractions was performed by detection of the raft marker ganglioside GM1 in 2 μl of each fraction spotted on nitrocellulose by HRP-labelled cholera toxin subunit B (1 h; Sigma, Germany). Signal strength was detected using the ECL Plus Western Blotting Detection System (GE Healthcare, Buckinghamshire, UK).

### Lipid extraction and high-performance thin layer chromatography (HPTLC)

Both methods were performed according to Grether-Beck et al. [23]. Briefly, quantification of lipids was carried out using 500 mg protein for Folch extraction. Ceramides need to be extracted from the biological sample by alkaline hydrolysis, whereas sphingomyelin and glycosphingolipids are extracted without the hydrolysation. After extraction, samples and standards were separated on silica gel HPTLC plates using a stepwise elution gradient with methanol, dichloromethane, and n-hexane. Visualization of separated bands was carried out by post-chromatic derivatisation after dipping in a manganese chloride solution.

### Western blotting

Cells: After exposure, cells were washed twice with ice-cold PBS and lysed in modified RIPA buffer [25 mM Tris/HCl, pH 7.4, 150 mM NaCl, 0.1 mM EDTA, 1% Nonidet P-40, 0.1% SDS, 1% deoxycholate, 0.025% NaN_3_, 1% protease inhibitor cocktail, and 1% phosphatase inhibitor cocktail]. Lung tissue: Minced tissue was incubated with RIPA buffer and protein was isolated using a pebble mill. After determination of protein contents, equal amounts of total cell protein [5 – 40 μg] were separated by SDS-PAGE [7.5 or 10%] and transferred onto PVDF membranes (Hybond-P, Amersham Biosciences, Little Chalfont, UK). The following antibodies were used: phospho-EGFR (Tyr^1173^), phospho SFK (Tyr^416^), and phospho-p44/42 MAPK (Thr^202^/Tyr^204^) (all from Cell Signalling Technology, Danvers, MA), total EGFR (Upstate Biotechnology, Lake Placid, NY), total SFK (Cell Signalling Technology), and GAPDH (Imgenex Corp., San Diego, CA). Signal strength was detected using the ECL Plus Western Blotting Detection System. Band intensities from X-ray films (immunosignal) were used for statistical calculations. The depicted graphs show immunosignals realtive to the respective controls.

### Immune staining

Cells were fixed with 4% paraformaldehyde for 15 min at room temperature. After permeabilisation and blocking (in 3% bovine serum albumin, 0.3% Triton X-100 in PBS), cells were incubated with anti-EGFR antibody (1:50; Cell Signalling Technology) overnight at 4°C. After incubation with anti-rabbit Alexa Fluor 594-coupled antibody (1 h, 1:500; Invitrogen, Darmstadt, Germany), nuclei were counterstained with 4′,6-diamidino-2-phenylindole (1:2000, Invitrogen). Cells were visualized using an Axiovert 40 microscope (Zeiss, Jena, Germany). As control for the specificity of the reactions, mock immunostainings without primary antibody were performed.

### ROS detection

Cells were incubated for dye uptake with 20 μM of the fluorogenic probe H_2_DCF-DA (2', 7'-Dichlorofluorescein diacetate, Calbiochem, Schwalbach, Germany). Generation of ROS was determined by flow cytometry according to Weissenberg et al. [8].

### Statistical analyses

In vitro experiments were repeated independently at least three times; animal experiments were performed with 5 animals per group. Unless otherwise stated, results were analyzed by analysis of variance followed by post-hoc testing. Differences between groups were considered as significant when p < 0.05.

## Competing interests

KU received research grants from bitop AG which is the producer of ectoine. JK acts as a consultant for bitop AG. None of the other authors has any financial competing interest.

## Authors’ contributions

HP together with US and DS designed and performed lipid raft extraction experiments and protein measurements. US designed all in vitro experiments and performed DCF flourescence measurements as well as protein detections. SG-B together with IF designed and performed lipid analysis experiments. SJ and JH designed and performed immunoflurescence staining experiments and microscopical analyses. MK and KU designed and performed the animal experiments. MG and DS performed physico-chemical analyses of the particle suspensions. CB performed WST and DCF measurements. JK contributed conceptually to the experiments with compatible solutes. KU is the author of the manuscript. All authors read and approved the final manuscript.

## Supplementary Material

Additional file 1 Exposure of cells to CNP or CP does not interfere with viability measured by WST. Click here for file
