# Supplementary material for: Carbon nanoparticles induce ceramide- and lipid raft-dependent signalling in lung epithelial cells: a target for a preventive strategy against environmentally-induced lung inflammation
Source: Part Fibre Toxicol. 2012 Dec 10;9:48. doi: 10.1186/1743-8977-9-48 (PMC3546038; doi:10.1186/1743-8977-9-48)
Supplement: Additional file 1 — Exposure of cells to CNP or CP does not interfere with viability measured by WST. [file 1743-8977-9-48-S1.doc]

Supplementary Material

Carbon nanoparticles induce ceramide- and lipid raft-dependent signalling in lung epithelial cells: a preventive strategy against environmental lung inflammation

Henrike Peuschel^1§^, Ulrich Sydlik^1§^, Susanne Grether-Beck^1^, Ingo Felsner^1^, Daniel Stöckmann^1^, Sascha Jakob^1^, Matthias Kroker^1^, Judith Haendeler^1^, Marijan Gotić^2^, Christiane Bieschke^1^, Jean Krutmann^1^ and Klaus Unfried^1#^

(^1^)IUF Leibniz Research Institute for Environmental Medicine, Düsseldorf, Germany, (^2^) Department of Material Chemistry Institute Ruđer Bosković, Zagreb, Croatia. (^§^) authors contributed equally. (^#^) corresponding author.

**supplementary Figure 1: Exposure of cells to CNP or CP does not interfere with viability measured by WST**

RLE-6TN cells were exposed for 1 h to the indicated doses (µg/cm^2^) of CNP or CP, respectively. Experiments were performed as independent triplicates (n=3). Depicted are means and standard deviations.

**Identification of lipid raft fractions**

Prior to analyses of raft-dependent signalling processes, lipid rafts were identified by GM1 staining. The possible influence of cell treatments with particles, ceramide, and ectoine on raft integrity was also controlled. As shown in figure 1A, raft fractions (1 and 2) were characterized by an enrichment of glanglioside GM1, while in the non-raft fractions (3 – 7) increasing amounts of the cytoplasmatic marker protein glycerinaldehyde 3-phosphate dehydrogenase (GAPDH) were found. None of the cell treatments, neither with CNP, non-nano carbon particles (CP), CNP with ectoine, nor with C6 significantly changed these marker patterns (figure 1B). Therefore, raft and non-raft fractions from each individual gradient were pooled for further analyses. The protein content of the gradient fractions showed also no significant differences between CNP treated and control treated lung epithelial cells (figure 1C).

**supplementary Figure 2: C6 as well as particle treatment does not disrupt raft structures**

Membrane fractions were isolated and raft-fractions (GM1 positive, GAPDH negative) were discriminated from non raft-fractions (GM1 negative, GAPDH positive) as described in materials and methods. A: Dot blot and respective densitometric analysis of membrane compartments of untreated RLE-6TN cells. *, Significantly different to raft-fraction 1. B: Identification of raft fractions by GM1 and GAPDH detection in cells treated with CNP [10 μg/cm^2^, 5 min], CP [10 μg/cm^2^, 5 min], ectoine [E; 1 mM, 4 h pre-treatment] + CNP, or C6 [5 µM, 15 min] treated RLE-6TN cells. C: Total protein content of isolated membrane fractions of control- and CNP- [10 μg/cm^2^, 5 min] treated RLE-6TN cells.

**Table 1:**

Table 1: Physical characteristics of carbon particles and particle suspensions

| sample (33µ/ml) | primary particle  size (SEM) | BET m^2^/g | cluster size (PBS) | zeta potential  (PBS) |
| --- | --- | --- | --- | --- |
| CNP | 20 nm | 442 | 5720 nm (16.1%)  1233 nm (16.5 %)  669 nm (37.6 %)  344 nm (29.8 %) | - 4.5 mV |
| CP | 350 nm | 10.6 | 1155 nm (39.7 %)  498 nm (52.8 %)  162 nm (7.5 %) | - 3.9 mV |
| CNP with 1 mM ectoine | n.d.* | n.d. | 5950 nm (7.9 %)  3190 nm (6.4 %)  1879 nm (20.7 %)  733 nm (12.6 %)  330 nm (52.4 %) | - 4.5 mV |
| CNP with 1 µg/ml BSA | n.d. | n.d. | 2141 nm (15.6 %)  743 nm (18.9 %)  471 nm (38.2 %)  214 nm (29.3 %) | -12.72 mV |

* not determined
